# Do Diabetes and Genetic Polymorphisms in the *COMT* and *OPRM1* Genes Modulate the Postoperative Opioid Demand and Pain Perception in Osteoarthritis Patients After Total Knee and Hip Arthroplasty?

**DOI:** 10.3390/jcm14134634

**Published:** 2025-06-30

**Authors:** Alina Jurewicz, Agata Gasiorowska, Katarzyna Leźnicka, Agnieszka Maciejewska-Skrendo, Maciej Pawlak, Anna Machoy-Mokrzyńska, Andrzej Bohatyrewicz, Maciej Tarnowski

**Affiliations:** 1 Department of Specialistic Nursing, Pomeranian Medical University, Żołnierska 48, 71-210 Szczecin, Poland; alina.jurewicz@pum.edu.pl; 2 Faculty of Psychology in Wroclaw, SWPS University, Ostrowskiego 30b, 54-238 Wroclaw, Poland; agasiorowska@swps.edu.pl; 3 Faculty of Physical Culture, Gdansk University of Physical Education and Sport, ul. K.Górskiego 1, 80-336 Gdansk, Poland; agnieszka.maciejewska-skrendo@awf.gda.pl; 4 Institute of Physical Culture Sciences, University of Szczecin, Al. Piastów 40b, 70-453 Szczecin, Poland; 5 Department of Physiology and Biochemistry, Poznan University of Physical Education, 61-871 Poznan, Poland; pawlak@awf.poznan.pl; 6 Department of Experimental and Clinical Pharmacology, Pomeranian Medical University, al. Powstanców Wlkp.72, 70-111 Szczecin, Poland; anna.machoy-mokrzynska@pum.edu.pl; 7 Department of Orthopaedics, Traumatology and Musculoskeletal Oncology, Pomeranian Medical University, Unii Lubelskiej 1, 71-252 Szczecin, Poland; bohatyrewicz@orthopedics.pl; 8 Department of Physiology in Health Sciences, Pomeranian Medical University, al. Powstanców Wlkp.72, 70-111 Szczecin, Poland

**Keywords:** *COMT*, *OPRM1*, polymorphism, pain, postoperative treatment, opioids, diabetes

## Abstract

**Background:** Osteoarthritis (OA) of the hip and knee is a common age-related degenerative disease characterized by joint pain, stiffness, and gait disturbances. This study investigated the influence of genetic polymorphisms in the *OPRM1* (rs1799971) and *COMT* (rs4633, rs4680, rs4818, and rs6269) genes on the postoperative analgesic requirements in 195 diabetic and non-diabetic patients undergoing total hip or knee arthroplasty. **Methods:** The prospective study included all patients who were admitted between January and September 2020 and agreed to participate. Postoperative pain management was assessed based on acetaminophen, ketoprofen, and morphine consumption on the first and second postoperative day. **Results:** Multilevel regression analyses revealed a significant three-way interaction between diabetes, type of analgesic, and *OPRM1*rs1799971 polymorphism, indicating different analgesic dosing patterns in diabetic and non-diabetic patients. Two-way interactions between diabetes and *COMT* polymorphisms rs4633, rs4680, and rs6269 further influenced the analgesic requirements. No significant associations were found for *COMT* rs4818. The results show that diabetes and genetic factors significantly influence opioid requirements and pain perception. **Conclusions:** Given the complexity of pain management in diabetic patients, personalized analgesic strategies tailored to genetic and metabolic profiles could be useful in postoperative pain management and reducing opioid consumption.

## 1. Introduction

Osteoarthritis (OA) of the hip and knee is a common age-related, progressive degenerative disease characterized by joint pain, stiffness, and gait disturbances. At the tissue level, the pathophysiology of OA includes osteophyte growth, cartilage degradation, synovial hyperplasia, abnormal joint structure, increased vascularity, and synovial inflammation. Ineffective treatment leads to the significant limitation of mobility and even disability. The progression of the disease, which manifests itself in the degeneration of articular cartilage, is related to joint incongruity, overuse, mechanical stress, and aging [1,2]. In addition, obesity, inflammation, diabetes, and lifestyle factors such as dynamic overloads from sports activities or, in contrast, static loads from sedentary activities contribute to the pathomechanism of OA [3,4,5,6]. In addition, the genetic background contributes significantly to the development and progression of OA [7].

The most common manifestation of the disease is joint pain. However, the cause of pain in osteoarthritis is certainly multifactorial, including oxidative stress, inflammation of the synovial membrane (synovitis), and dysregulation of the immune system [8]. In addition, limited joint mobility, joint stiffness, instability, swelling, and muscle weakness or atrophy are specific features of OA [9]. Despite the considerable efforts and progress in the treatment of the disease, there is still no effective cure in sight. The available treatment options focus on reducing pain, improving joint function, and increasing quality of life and can be categorized as follows: basic treatment (such as weight reduction and physical activity), medication, and surgery. The relief of symptomatic pain is usually based on a pharmacological approach and the use of anti-inflammatory non-steroidal anti-inflammatory drugs (NSAIDs), glucocorticoids, or acetaminophen [10].

Common antipyretic analgesics such as acetaminophen and both antipyretic and anti-inflammatory analgesics such as ketoprofen provide effective pain relief [11,12]. NSAIDs relieve pain and inflammation by inhibiting cyclo-oxygenases (COX): COX-1 and COX-2. The effect of COX inhibitors is primarily based on a strong down-regulation of prostaglandin synthesis. COX-1 is an inherent housekeeping enzyme [13] that is mainly found in the stomach, kidney, and blood platelets. It catalyzes the production of the physiologically necessary prostaglandin E2 (PGE2), which regulates peripheral vascular resistance and platelet aggregation, maintains blood flow in the kidneys, and protects the gastric mucosa [14]. On the other hand, COX-2 is expressed by monocytes, macrophages, fibroblasts, etc., in response to inflammatory stimulation and is, therefore, referred to as an inducible enzyme [15]. While the inhibition of COX-2 reduces prostaglandin synthesis and relieves pain and swelling, it can also irritate the gastric mucosa, leading to nausea, vomiting, diarrhea, and other gastrointestinal symptoms. Numerous studies suggest that NSAIDs may act on central pain mechanisms by influencing widespread hyperalgesia [16,17,18].

If drug therapy fails, severe, persistent pain is usually an important indicator for joint replacement in the late stages of OA. Surgical treatment options for OA include arthroscopic surgery and total knee/hip arthroplasty (TKA/THA) [19]. Arthroplasty is considered the definitive solution for advanced OA, and the number of cases operated on is increasing worldwide [20,21]. After TKA, pain, function, and quality of life improve in the majority of patients compared to non-surgical treatments [22]. However, it is evident that 10–40% of patients still suffer from chronic postoperative pain after hip and knee replacement surgery [23,24]. In general, arthroplasty is a successful orthopedic procedure for the treatment of symptomatic knee/hip OA, reducing pain and improving function and quality of life. Arthroplasty leads to pain relief, improved function, and an overall higher quality of life.

The prevention and relief of postoperative pain is an important cornerstone in the care of surgical patients as it has a significant impact on morbidity, recovery, and overall quality of life [25]. Postoperative pain management aims to improve postoperative outcome, rehabilitation, quality of life, and patient satisfaction, and allows for early mobilization [26]. Consequently, effective postoperative analgesia is crucial for rapid recovery and discharge from hospital [27,28,29]. Opioids are most effective in the treatment of acute pain, including postoperative pain, but they are also associated with negative side effects such as nausea, vomiting, and drowsiness, as well as the risk of dependence [30]. Therefore, it makes sense to optimize the dose to achieve effective pain control and avoid excessive side effects. However, some patients respond better to these treatments than others and require individually adjusted doses of analgesics depending on the case and concomitant conditions, such as diabetes. Pain is a complex subjective phenomenon that is perceived differently by each person and is influenced by biological, psychological, and genetic factors. In addition, genetic variability can influence the perception of pain and the response to analgesic therapy [31].

Over the past two decades, various genetic approaches—including knockdown animal studies, twin studies, candidate gene approaches, and genome-wide analyses—have been used extensively to investigate the genetic basis of pain perception and the response to pharmacological treatment in adults [32,33]. Among the genetic factors studied, single-nucleotide polymorphisms (SNPs) in genes related to drug metabolism have received considerable attention. The most consistently validated genetic variants affecting the efficacy of morphine in patients are found in the genes encoding catechol-O-methyltransferase (COMT) and the μ-opioid receptor (OPRM1) [31,34]. The important functional variant A118G (rs1799971 polymorphism) of the *OPRM1* gene has been shown to influence the need for analgesics in chronic pain as well as pain management [35]. The enzyme COMT metabolizes catecholamines, including dopamine, epinephrine, and norepinephrine, and its activity would, therefore, influence the need for analgesics in pain management [36,37,38]. However, the available data on the association between the *COMT* and *OPRM1* genes and analgesic efficacy are very limited.

Diabetes impairs nerve function and affects nociceptive physiology, leading to increased hypersensitivity to pain and a weaker response to morphine [39], particularly in neuropathic pain [40]. According to one report, diabetic patients suffer more pain than non-diabetic patients both pre- and postoperatively [41]. Postoperative pain and analgesic requirements have been found to be significantly higher in diabetic patients with lower limb fractures [42]. In a recently published retrospective observational study, data from over 43,000 surgical patients were analyzed. It was found that patients with diabetes had higher preoperative pain scores and more frequent preoperative opioid use compared to non-diabetic patients [43]. In addition, diabetes is a risk factor for prolonged opioid use (POE) [44].

Building on this knowledge, the present study investigates how polymorphisms in the *OPRM1* and *COMT* genes are associated with analgesic requirements in diabetic and non-diabetic patients with osteoarthritis (OA) following total hip or knee arthroplasty.

## 2. Materials and Methods

### 2.1. Study Recruitment

The study cohort was recruited from the Department of Orthopedics, Traumatology and Musculoskeletal Oncology at the Pomeranian Medical University. Participants included all patients who were consecutively admitted to the clinic between January and September 2020, met the inclusion criteria, consented to the study, and were scheduled for surgery (195 out of 367 patients admitted—53.1%).

The inclusion criteria were as follows: (1) patients undergoing elective primary total hip arthroplasty and total knee arthroplasty, (2) due to osteoarthritis documented by radiologic assessment—moderate to severe radiologic changes (grade 3 and 4) according to the Kellgren and Lawrence classification [45], and (3) >18 years and ≤80 years.

Exclusion criteria included (1) patients ≤ 18 years and >80 years, (2) patients with rheumatoid arthritis, (3) patients admitted for secondary arthroplasty procedures—revisions, for post-traumatic arthritis, (4) with concomitant diseases potentially affecting the metabolism of analgesics—chronic hepatic insufficiency, uremia, (5) opioids, non-steroidal anti-inflammatory drugs, or other chronic pain medication’ users—defined as more than 2 times per week, and (6) any additional use of pain medication (off-protocol) prescribed by medical staff or medication taken from the individual patient’s resources during hospitalization

An uncemented Pinnacle^®^ cup and a Corail^®^ stem (DePuy Synthes, Warsaw, IN, USA) were used for the THR. The operations were performed by two experienced hip surgeons using the anterolateral approach as described by Watson-Jones [46]. For the TKR, a cemented Vanguard^®^ knee (Biomet, Inc., Warsaw, IN, USA) was implanted, with the procedures performed via the medial parapatellar approach [47] by two experienced knee arthroplasty surgeons.

All patients received midazolam (0.05 mg/kg) as premedication. Routine subarachnoid anesthesia with 0.5% bupivacaine (0.15 mg/kg) was administered after determination of the L2/L3 level, which is suitable for both hip and knee procedures [48]. The average hospital stay was 3.6 days and ranged from 3 to 5 days.

### 2.2. Participants

This prospective study involved 195 patients who underwent hip or knee replacement surgery, including 85 women and 110 men, with a mean age of 63.31 years (*SD* = 9.03, median = 65, range 33–77). Participants had an average BMI of 30.10 (*SD* = 4.74, median = 29.80, range 20.40–43.40), and 41 individuals (21.03%) were diagnosed with type 2 diabetes with regularly monitored blood glucose levels. A sensitivity analysis performed using Monte Carlo simulation in Mplus [49] confirmed that this sample size was sufficient to detect an effect size of f = 0.09 (η^2^ = 0.009) for the interaction between the person-level factors at a statistical power of 1 − β = 0.80 and a significance level of α = 0.05. Thus, the sample was sufficiently powerful to detect even weak associations between the variables of interest.

### 2.3. Procedure

The participants were fully informed about the purpose of the study and gave their written consent to participate. The research protocol was approved by the Bioethics Committee of the German Medical Association on 14 October 2019 (approval number KB–0012/163/19). Upon admission to the hospital, all participants confirmed that they were fasting and had not taken any painkillers recently (at least 24 h).

The analgesics were administered according to a single-blinded protocol based on a standardized pain management plan. Pain was assessed using a standardized numerical rating scale as described by Nugent at al [50]. Patients reporting mild pain (1–3 points) received 1 g of acetaminophen intravenously 1–4 times daily as needed [51]. For mild pain rated at 4 points, an additional 0.1 g of intravenous ketoprofen was prescribed 1–2 times daily [52]. Moderate pain (NRS > 4) was treated with 0.01 g intravenous morphine administered up to four times daily until pain intensity was reduced to 4 or less [53]. Patients and medical staff were repeatedly reminded not to exceed the pharmacologic protocol during hospitalization.

The primary outcome measure for postoperative pain management was the consumption of acetaminophen, ketoprofen, and morphine on the first and second postoperative day. All patients required analgesics on both days; 72.1% of the cohort (*n* = 142) received all three substances on the first day, and 16.9% (*n* = 33) received all three substances on the second day.

### 2.4. Genotyping

Genomic DNA was extracted from buccal cells using a Genomic Micro AX SWAB Gravity (A&A Biotechnology, Gdańsk, Poland) according to the manufacturer’s protocol. All samples were genotyped using allele discrimination assays with TaqMan^®^ probes (Applied Biosystems, Carlsbad, CA, USA) on a 7500 Fast Real-Time PCR Detection System (Applied Biosystems). Four SNPs were genotyped for the COMT1 gene—C/T rs4633, A/G rs4680, C/G rs4818, and A/G rs6269—while one SNP was genotyped in the *OPRM1* gene—A/G rs1799971. To discriminate between the *OPRM1*rs1799971 alleles, TaqMan^®^ Pre-Designed SNP Genotyping Assays (Applied Biosystems) (assay ID: C___8950074_1_), for COMT1 rs4633, rs4680, rs4818, and rs6269 (assays ID: C___2538747_20, C__25746809_50, C___2538750_10 and C___2538746_1_, respectively) consisting of fluorescently labelled (FAM and VIC) minor groove binder (MGB) probes and two specific primers, were used. All samples were genotyped in duplicate.

### 2.5. Statistical Analysis

The descriptive statistical analysis was performed with JAMOVI [54]. The threshold for statistical significance was set at *p* < 0.05. To test the Hardy–Weinberg equilibrium, we used a chi-square test.

The data in this study were considered nested, with six data points per participant (the doses for three analgesics taken over two days). Therefore, we used a multistep regression with Mplus 8.10 using Bayesian estimation, which allows the use of variables that deviate from the normal distribution and have non-continuous values [55]. In step 1, we regressed a dose of analgesics as DV (multilevel Z-score for each substance separately) on the presence of diabetes (yes vs. no), polymorphisms within the analyzed genes, the type of substance administered (acetaminophen vs. ketoprofen vs. morphine), and their interactions. In step 2, we added age and BMI as covariates to test whether the effects we found after controlling for these two variables were robust and significant. We performed these analyses six times, separately for each gene.

We assumed that our data were nested within participants, days, and types of operations. Therefore, we calculated ICCs for three possible random intercepts. The ICC for the random intercept for the postoperative day was relatively high, ICC_day_ = 0.27. At the same time, the ICCs for the other parameters were relatively low, namely, ICCID = 0.07, and ICC_surgery_ = 0, suggesting that only a very small proportion of the variance in DV was due to differences between these clusters. To summarize, we included a random intercept only for the postoperative day (first vs. second).

## 3. Results

### 3.1. Hardy–Weinberg Equilibrium

The genotype frequencies did not deviate from the Hardy–Weinberg expectations for all polymorphisms, namely, for *COMT* rs4633: CC 25.6%, CT 45.1%, TT 29.2%, χ2(1) = 1.81, *p* = 0.405; for *COMT* rs4680: GG 25.1%, AG 45.6%, AA 29.2%, χ2(1) = 1.43, *p* = 0.489; for *COMT* rs4818: CC 39.5%, CG 48.7%, GG 11.8%; χ2(1) = 0.60, *p* = 0.742; for *COMT* rs6269: AA 39.5%, GA 48.7%, GG 11.8%, χ2(1) = 0.60, *p* = 0.742; and for *OPRM1*rs1799971: AA 80.9%, AG 17.5%, GG 1.5%, χ2(1) = 0.53, *p* = 0.767.

### 3.2. Descriptive Analyses and Correlations

Table 1 shows the descriptive statistics for the whole sample and compares the two types of surgery. We did not find significant differences between participants undergoing hip and knee surgery in terms of age and dose of analgesics administered. The only significant difference was the BMI: patients who underwent hip surgery had a lower BMI than those who underwent knee surgery. The proportion of men and women did not differ by type of surgery, χ2(1) = 1.56, *p* = 0.212. The proportion of patients with diagnosed diabetes, χ2(1) = 1.25, *p* = 0.263, and their mean age also did not differ significantly (see Table 1).

Table 2 shows the correlations between the variables of interest. We found that older participants and participants with a higher BMI were more likely to be diagnosed with diabetes than younger participants and participants with a lower BMI. The administration of higher doses of acetaminophen on the first postoperative day was associated with the administration of higher doses of ketoprofen and morphine on that day. However, the doses of the latter two analgesics were not correlated. Higher doses of acetaminophen on the first postoperative day were also associated with higher doses of acetaminophen and ketoprofen on the second postoperative day.

### 3.3. Multilevel Regression Analysis

#### 3.3.1. *OPRM1*rs1799971 Polymorphisms

Diabetes, substance, polymorphisms of *OPRM1*rs1799971 (AA vs. AG/GG), and their interaction explained R^2^ = 2.7%, *p* = 0.001 of the variance in the dose of analgesics. As shown in Table 3, none of the main effects and none of the two-way interactions were significant. However, we found a significant three-way interaction between the type of analgesic (morphine vs. acetaminophen), diabetes, and the polymorphisms in *OPRM1*rs1799971. This interaction remained significant when we controlled for age and BMI in the second step of the analysis (see Table 3).

**Figure 1:**
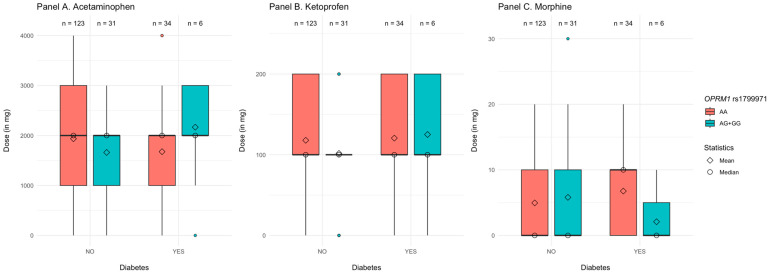
Dose of the analgesics received by patients with and without diabetes as a factor of the type of substance and *OPRM1*rs1799971 polymorphisms.

We further decomposed this interaction by examining the two-way interactions between the *OPRM1*rs1799971 polymorphisms and the type of analgesic administered separately for patients with and without diabetes. In patients without diabetes, the main effect of the analgesic (morphine vs. acetaminophen) was not significant, β = 0.04, post *SD* = 0.05, 95% CI [−0.05, 0.14], *p* = 0.400, same as the main effect of the *OPRM1*rs1799971 polymorphisms, β = −0.08, post *SD* = 0.04, 95% CI [−0.15, 0.01], *p* = 0.080. However, the two-way interaction between these two factors was significant, β = 0.11, post *SD* = 0.05, 95% CI [0.01, 0.20], *p* = 0.040. Further decompositions demonstrated that, while participants with AG/GG alleles did not differ from those with AA alleles regarding the dose of morphine, β = 0.05, post *SD* = 0.07, 95% CI [−0.08, 0.20], *p* = 0.380, they received lower doses of ketoprofen, β = −0.16, post *SD* = 0.07, 95% CI [−0.28, −0.001], *p* = 0.040, and lower doses of acetaminophen, β = −0.18, post *SD* = 0.07, 95% CI [−0.30, −0.02], *p* = 0.020 (see Figure 1 and Table S1 in Supplementary Materials).

In patients with diabetes, the main effect of the analgesic was not significant, β = −0.11, post *SD* = 0.11, 95% CI [−0.29, 0.11], *p* = 0.360, same as the main effect of the *OPRM1*rs1799971 polymorphisms, β = −0.02, post *SD* = 0.09, 95% CI [−0.18, 0.12], *p* = 0.700. Once more, the two-way interaction was significant, β = −0.33, post *SD* = 0.11, 95% CI [−0.50, −0.09], *p* < 0.001. Further decompositions demonstrated that the pattern in this interaction was different from that of patients without diabetes. Participants with AG/GG alleles received lower doses of morphine than those with AA, β = −0.39, post *SD* = 0.18, 95% CI [−0.67, −0.001], *p* = 0.040. At the same time, we did not find a significant difference regarding the doses of acetaminophen, β = 0.26, post *SD* = 0.18, 95% CI [−0.04, 0.65], *p* = 0.100, and the doses of ketoprofen, β = 0.03, post *SD* = 0.18, 95% CI [−0.28, 0.41], *p* = 0.800 (see Figure 1).

#### 3.3.2. *COMT* rs4633 Polymorphisms

Diabetes, substance, polymorphisms of *COMT* rs4633 (CC vs. CT vs. TT), and their interaction explained R^2^ = 3.0%, *p* = 0.001 of the variance in the dose of analgesics. As shown in Table 4, the only significant main effect for *COMT* was rs4633 polymorphisms, such that participants with the CT allele received higher doses of analgesics than participants with the CC allele. We also found a significant two-way interaction between diabetes and (CT vs. CC) polymorphisms in *COMT* rs4633. The main effect of *COMT* rs4633 and the above-mentioned interaction remained significant when we controlled for age and BMI in the second step of the analysis. The three-way interaction between diabetes, polymorphisms in *COMT* rs4633, and type of substance was not significant, suggesting that the interaction between diabetes and *COMT* rs4633 polymorphisms was similarly associated with morphine, ketoprofen, and acetaminophen use.

**Figure 2:**
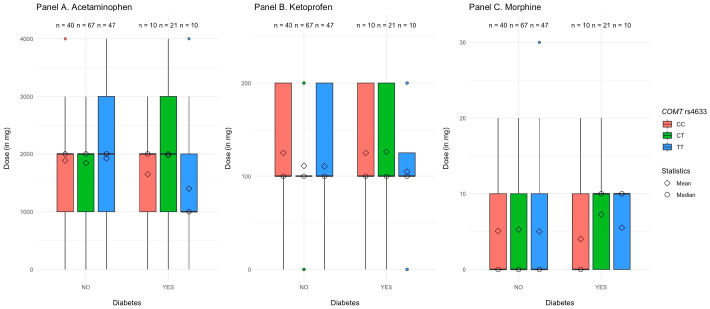
Dose of the analgesics received by patients as a factor of their diabetes and *COMT* rs4633 polymorphisms.

We further decomposed the two-way interaction by examining the differences between patients with CC vs. CT vs. TT alleles in the *COMT* rs4633 gene separately for patients with and without diabetes. We found that, in patients without diabetes, the effect of the CT vs. TT *COMT* rs4633 polymorphisms was not significant, β = −0.01, Post *SD* = 0.04, 95% CI [−0.08, 0.07], *p* = 0.780. However, among patients with diabetes, those with the CT allele received higher doses of analgesics when compared to those with the TT allele, β = 0.23, Post *SD* = 0.08, 95% CI [0.05, 0.34], *p* = 0.020. There was no significant difference for the doses of analgesics between participants with CC and TT alleles, no matter if they were diagnosed with diabetes, β = 0.05, Post *SD* = 0.10, 95% CI [−0.23, 0.19], *p* = 0.720, or not, β = 0.02, Post *SD* = 0.04, 95% CI [−0.05, 0.11], *p* = 0.480. An alternative decomposition of the same interaction revealed that participants with CC and TT alleles received similar doses of analgesics irrespective of whether they had diabetes or not, respectively, β = −0.10, Post *SD* = 0.07, 95% CI [−0.22, 0.04], *p* = 0.160 for CC alleles, and β = −0.05, Post *SD* = 0.07, 95% CI [−0.22, 0.06], *p* = 0.380 for TT alleles. However, participants with the CT allele required higher doses of analgesics when they had diabetes vs. not, β = 0.13, Post *SD* = 0.05, 95% CI [0.03, 0.22], *p* < 0.001 (see Figure 2 and Table S2 in Supplementary Materials).

#### 3.3.3. *COMT* rs4680 Polymorphism

Diabetes, substance, polymorphisms of *COMT* rs4680 (AA vs. AG vs. GG), and their interaction explained R^2^ = 3.3%, *p* = 0.001 of the variance in the dose of analgesics. As demonstrated in Table 5, the only significant main effect was the one of the *COMT* rs4680 polymorphisms, such that participants with the AG allele received higher doses of analgesics than those with the AA allele. Additionally, we found a significant two-way interaction between diabetes and AG vs. AA polymorphisms in *COMT* rs4680. The main effect of *COMT* rs4680 and the aforementioned interaction remained significant when we controlled for age and BMI in the second step of the analysis. The three-way interaction between diabetes, polymorphisms in *COMT* rs4680, and the type of substance was not significant, indicating that the interplay between diabetes and *COMT* rs4680 polymorphisms was associated similarly with doses of morphine, ketoprofen, and acetaminophen.

We further decomposed the two-way interaction by investigating the differences between patients with AA vs. AG vs. GG alleles in the *COMT* rs4680 gene separately for patients with and without diabetes. We found that, in patients without diabetes, the effect of the AG vs. AA *COMT* rs4680 polymorphisms was not significant, β = −0.01, Post *SD* = 0.04, 95% CI [−0.07, 0.07], *p* = 0.820. However, among patients with diabetes, those with the AG allele received higher doses of analgesics when compared to those with the AA allele, β = 0.22, Post *SD* = 0.08, 95% CI [0.04, 0.33], *p* = 0.020. There was no significant difference for the doses of analgesics between participants with GG and AA alleles, no matter if they were diagnosed with diabetes, β = 0.04, Post *SD* = 0.10, 95% CI [−0.23, 0.18], *p* = 0.700, or not, β = 0.02, Post *SD* = 0.04, 95% CI [−0.06, 0.11], *p* = 0.560.

**Figure 3:**
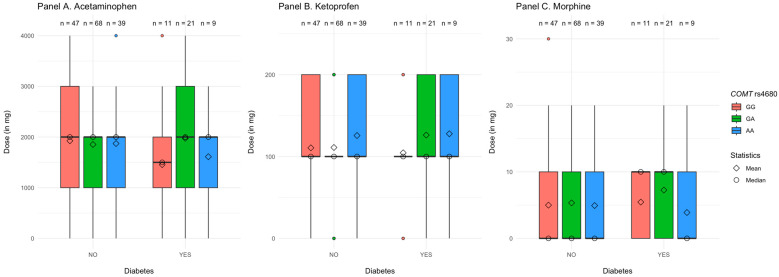
Dose of the analgesics received by patients as a factor of their diabetes and *COMT* rs4680 polymorphisms.

An alternative decomposition of the same interaction revealed that participants with AA and GG alleles received similar doses of analgesics irrespective of whether they had diabetes or not, respectively, AA: β = −0.10, Post *SD* = 0.07, 95% CI [−0.20, 0.04], *p* = 0.180 and GG: β = −0.05, Post *SD* = 0.07, 95% CI [−0.23, 0.06], *p* = 0.460. However, participants with the AG allele required higher doses of analgesics when they had diabetes vs. not, β = 0.12, Post *SD* = 0.05, 95% CI [0.03, 0.22], *p* < 0.001 (see Figure 3 and Table S3 in Supplementary Materials).

#### 3.3.4. *COMT* rs4818

Diabetes, substance, polymorphisms of *COMT* rs4818 (CC vs. CG vs. GG), and their interaction explained less than R^2^ = 2.3%, *p* < 0.001 of the variance in the dose of analgesics. However, as demonstrated in Table 6, none of the effects were significant. We did not find any significant effects when we controlled for age and BMI in the second step of the analysis.

#### 3.3.5. *COMT* rs6269

Diabetes, substance, polymorphisms of *COMT* rs6269 (AA vs. AG vs. GG), and their interaction explained R^2^ = 2.5%, *p* < 0.001 of the variance in the dose of analgesics. As demonstrated in Table 7, the only significant effect was a two-way interaction between diabetes and GG vs. AA polymorphisms in *COMT* rs4680. It remained significant when we controlled for age and BMI in the second step of the analysis.

We further decomposed the two-way interaction by investigating the differences between patients with AA vs. AG vs. GG alleles in the *COMT* rs6269 gene separately for patients with and without diabetes. We found that, in patients without diabetes, the effect of the GA vs. AA *COMT* rs6269 polymorphisms was not significant, β = −0.02, Post *SD* = 0.03, 95% CI [−0.09, 0.05], *p* = 0.740, same as for the patients with diabetes, β = −0.02, Post *SD* = 0.03, 95% CI [−0.09, 0.05], *p* = 0.740. At the same time, the effect of GG vs. AA *COMT* rs6269 polymorphisms was significant for the patients with diabetes, β = −0.24, Post *SD* = 0.11, 95% CI [−0.46, −0.03], *p* < 0.001, but not for those without diabetes, β = 0.07, Post *SD* = 0.05, 95% CI [−0.02, 0.17], *p* = 0.140.

**Figure 4:**
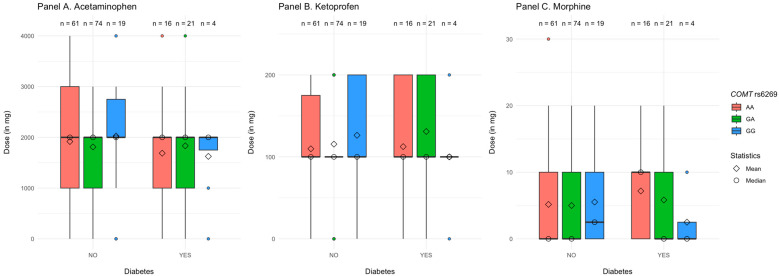
Dose of the analgesics received by patients as a factor of their diabetes and *COMT* rs6269 polymorphisms.

An alternative decomposition of the same interaction revealed that participants with AA and GA alleles received similar doses of analgesics irrespective of whether they had diabetes or not, respectively, β = 0.01, Post *SD* = 0.06, 95% CI [−0.10, 0.14], *p* = 0.900, and β = 0.06, Post *SD* = 0.05, 95% CI [−0.22, 0.18], *p* = 0.160. However, participants with the GG allele required lower doses of analgesics when they had diabetes vs. not, β = −0.24, Post *SD* = 0.09, 95% CI [−0.41, 0.06], *p* < 0.001 (see Figure 4 and Table S4 in Supplementary Materials).

## 4. Discussion

Acute postoperative pain is a common challenge in clinical practice and can lead to a number of consequences if not properly managed. One of the most serious consequences is the development of chronic postoperative pain. The use of drugs that act on different mechanisms of nociception offers benefits in terms of additive or synergistic effects and also helps to reduce the risk of adverse effects. Systemic analgesics are still the most commonly used method of pain relief for acute pain. The choice of opioids is based on their physicochemical properties and their ability to penetrate the brain. Published studies confirm that both the pharmacokinetics and pharmacodynamics of various drugs can be significantly altered in people with diabetes [56]. Drug absorption can be impaired by diabetes-related changes in blood flow to subcutaneous adipose tissue, skeletal muscle, and delayed gastric emptying. In addition, the distribution of drugs can be impaired by the non-enzymatic glycation of plasma proteins such as albumin, which alters the binding of drugs and, thus, their distribution in the body. The biotransformation or metabolic processing of drugs can also be affected by diabetes, through changes in the regulation of liver enzymes and transporters involved in drug metabolism. Finally, the excretion of drugs may be impaired as a result of diabetic nephropathy, which impairs kidney function and alters the elimination of drugs from the body. These combined factors highlight the importance of taking diabetes-related physiological changes into account when determining appropriate drug dosing and therapeutic strategies for diabetic patients. In clinical practice, however, a key element of opioid therapy is its high concentration relative to its analgesic effect. Scientific evidence suggests that effective pain relief with non-opioid medications relies on the use of paracetamol in combination with NSAIDs. There is growing evidence that genetic factors contribute significantly to individual differences in pain perception and are associated with an increased risk of developing chronic pain conditions. Scientists are still trying to understand the genetic basis of pain phenotypes and the efficacy of pain therapies in order to develop more precise treatments tailored to individual patient characteristics [57]. They are trying to achieve the best therapeutic results while minimizing side effects. The current research is focused on, among other things, developing models to predict pain medication dosing based on genetic polymorphisms that could be used in clinical practice [58], as well as developing tools for rapid genotyping in the hospital [59]. Although personalized treatment is the future of medicine, it is becoming particularly important in the treatment of pain conditions due to the large individual variability in pain sensitivity [60,61,62].

The search for genetic aspects of pain has led to the selection of several candidate genes. *COMT* and *OPRM1* are considered as potential “pain genes” because their products are functionally related to pain sensitivity and analgesia.

The μ-opioid receptor plays a key role in the action of endogenous and exogenous analgesic substances such as β-endorphin, enkephalin, and morphine. This is of great importance for the physiological and psychological response of the organism to stress, trauma, and pain [63]. The A118G polymorphism (functional substitution A > G at locus 118; rs1799971) is one of the most frequently studied single-nucleotide polymorphisms (SNPs) in the *OPRM1* gene. This variant affects the potential glycosylation site and stability of the μ-opioid receptor protein, reducing its expression and signaling [64,65]. The rs1799971 polymorphism has been shown to influence the need for analgesics in chronic pain as well as pain perception and treatment [66,67].

Several studies and meta-analyses show that carriers of the *OPRM1* (A118G) G allele require higher opioid doses to achieve analgesia [35]. The AA homozygous patients experience a lower pain intensity when treated with morphine compared to GG homozygous patients [68,69]. However, the results are inconsistent, which could be due to a different response in relation to the ligand (opioid drug) of the receptor [70,71]. Similarly, the G allele has been shown to be associated with postoperative pain [72,73,74,75]. On the other hand, these mutant homozygous patients were associated with a lower incidence of nausea compared to *OPRM1* wild types (A118A) [73,74]. Interestingly, both mutation heterozygotes and homozygotes were found to respond better to tramadol [75].

In our study, we found that participants without diabetes with AG/GG alleles did not differ from those with AA alleles in terms of morphine dose, but they received lower doses of ketoprofen (*p* = 0.033) and lower doses of acetaminophen (*p* = 0.017). Interestingly, patients with diabetes and with AG/GG alleles received lower doses of morphine than patients with AA (*p* = 0.013). We found no significant difference in the doses of ketoprofen and acetaminophen.

To our knowledge, there are no studies in the literature showing the association between the *OPRM1* (A118G) polymorphism and diabetes. However, the SNP could have an effect on ischemic pain, as found in studies on diabetic foot ulcers [76] and ischemic pain [77]. It has been shown that patients with the A118G variant endure more pressure pain and ischemic pain, presumably due to an increased sensitivity to endogenous opioids [78]. Epidural opioids are commonly used for analgesia in women in labor. Women who are heterozygous or homozygous for the *OPRM1* A118G allele have been shown to have a higher pressure pain threshold than women who are homozygous for the more common A allele [77]. The nucleotide substitution A/G leads to an amino acid switch from asparagine to aspartic acid and is thought to result in a higher binding affinity of β-endorphin to the opioid μ-receptor. In another study of women in labor, women with the A/A genotype required a higher intrathecal dose of fentanyl to achieve effective analgesia compared to women who were heterozygous or homozygous for the G allele [79,80]. These contradictory results emphasize the complexity of pain management.

The *COMT* gene is polymorphic, and the most frequently studied variants include rs4680, rs6269, rs4633, and rs4818. Three common SNPs in the *COMT* gene—rs4633, rs4680, and rs4818—are located in the central coding region of both the membranous and soluble forms of COMT (S-COMT and MB-COMT, respectively). The fourth SNP rs6269 is located in the promoter region and, together (a system of four SNPs in the *COMT* gene), forms a haplotype. The gene encodes a key enzyme involved in the metabolism of catecholamines such as adrenaline and noradrenaline and in the inactivation of dopamine. This enzyme is involved in numerous psychological and physiological processes, including pain modulation [37,38]. The most commonly studied variant in this gene is rs4680, which, unlike the other three, is non-synonymous and results in a substitution of guanosine (G) for adenosine (A) at codon 158. The *COMT* variant Val158Met influences enzyme activity, neurotransmitter levels, and pain threshold, and affects pain perception in subjects [81,82]. Previously published studies suggest that the *COMT* SNP rs4680 is associated with pain and opioid use, but not all data presented can provide convincing conclusions. It has been shown that a mutant homozygous rs4680 genotype is associated with higher pain scores [83] and lower opioid use [79,82].

A recent meta-analysis found that the presence of the rs4680-A allele is significantly associated with an increased incidence of chronic postoperative pain scores and that there is no significant difference in pain scores or opioid consumption in carriers of the rs4680 allele in the acute postoperative setting in either dominant or recessive inheritance models [72].

The mutant G allele of rs4818 has also been associated with higher pain scores [84] and homozygous mutants (G/G) were found to have a lower opioid consumption in the postoperative period [83]. In addition, Rakvag and colleagues found that the rs4818 genotype was associated with variations in the amount of opioid consumption in Caucasian cancer patients, with carriers of the GG genotype consuming more opioids [85].

Interestingly, our results suggest that, similar to the *OPRM1*rs1799971 polymorphism, diabetes is an important factor contributing to analgesic intake. We found insignificant effects of *COMT* rs4680, rs4633, rs6269, and rs4818 in patients without diabetes. The diabetic study participants with the AG allele of rs4680 received higher analgesic doses compared to those with the AA allele (*p* = 0.005), while participants with AA and GG alleles received similar analgesic doses; participants with the AG allele required higher analgesic doses if they had diabetes compared to those without diabetes (*p* = 0.021). In the case of the *COMT* rs4633 polymorphism, diabetics with the CT allele received higher analgesic doses than those with the CC allele (*p* = 0.005), and carriers of the CC and TT alleles received similar analgesic doses; participants with the CT allele required higher analgesic doses if they had diabetes than those without diabetes (*p* = 0.018). An analysis of another *COMT* rs6269 polymorphism revealed that patients with AA and GA alleles with or without diabetes received similar doses of analgesics. However, participants with diabetes and GG alleles required lower doses of analgesics compared to non-diabetics. We found no significant effects for the rs4818 polymorphism in the *COMT* gene.

However, the three combinations of *COMT* SNPs rs6269, rs4633, rs4818, and rs4680 result in three haplotypes of pain sensitivity, designated LPS (GCGG), APS (ATCA), and HPS (ACCG), which are decoded as low, medium, and high pain sensitivity, respectively. The carriers of a certain haplotype are characterized by a different sensitivity to experimental pain [49]. Pain sensitivity haplotypes have been attributed to differences in *COMT* activity, with the LPS haplotype having 4.8-fold-higher activity than the APS and 11.4-fold that of the HPS haplotype [49].

Our results indicate an influence of genetics and diabetes on the need for analgesics. The amount of available data on the association between diabetes and polymorphisms in the “pain genes” (*COMT* and *OPRM1*) is very limited. One study found that the *COMT* rs4680 G allele was associated with a lower HbA1c and provided modest protection against T2DM [86], while another study showed that the presence of one or two A alleles of *COMT* Val108/158Met was associated with an improved glycemic response and a better response to insulin therapy [87].

While studies have independently examined the effects of diabetes and genetic polymorphisms on postoperative opioid use, there are few studies that specifically address their combined effects. As both diabetes and genetic factors can influence pain perception and opioid metabolism, future research should aim to investigate how these variables interact. Understanding these relationships could lead to more individualized and effective pain management strategies for postoperative patients.

Our study encompassed patients who underwent uncomplicated TKR and THR. The decision to join both procedures together in one study is based on the publications underlining that the magnitude of postoperative pain intensity after THR and TKR does not differ significantly [48].

Our study has several limitations that must be acknowledged. First, the study was conducted at a single center. The considerable number of excluded patients further reduced the sample size and limited the generalizability of our results. Second, the data on pain medication use were collected before hospitalization from patients who sometimes did not remember which medications they had taken. Third, whereas the patients with a second type of diabetes were included in the study, the duration of the disease was not determined. Therefore, the impact of persistent diabetic nerve damage on pain perception and postoperative analgesic requirements could not be assessed and requires further study. Fourth, the study protocol included drugs that are common analgesics—acetaminophen, non-steroidal drugs—ketoprophen, and opioids—morphine, which are recommended in standard pain management after total joint replacement in orthopedics. It is important to mention that there are a variety of NLPZ for postoperative treatment that need to be investigated in follow-up studies.

## 5. Conclusions

In conclusion, both diabetes and genetic polymorphisms in the *COMT* and *OPRM1* genes play an important role in postoperative opioid demand and pain perception. Diabetes complicates pain management; therefore, customized postoperative pain management strategies for diabetic patients are needed. Since the satisfying treatment of pain is still an unsolved clinical problem, any attempt to explain this phenomenon on a molecular basis seems reasonable. Further research is needed to understand their combined effects and to develop tailored pain management strategies that take into account both metabolic and genetic factors.

## Figures and Tables

**Table 1:** Descriptives for the variables measured in the study, along with comparisons across types of surgery.

Variable	Min	Max	M	Mdn	*SD*	Hip Replacement *n* = 133		Knee Replacement *n* = 62		Comparison Between Types of Surgery	
						M	*SD*	M	*SD*	t(193)	Cohen’s d
Age	33	77	63.31	65	9.03	62.73	9.78	64.56	7.07	1.32	0.20
BMI	20.4	43.4	30.1	29.8	4.74	29.18	4.39	32.08	4.90	4.14 ***	0.64
Morphine—first day (in mg)	0	20	8.67	10	5.9	8.46	5.75	9.11	6.24	0.72	0.11
Morphine—second day (in mg)	0	30	1.97	0	4.61	1.8	4.66	2.34	4.50	0.75	0.12
Ketoprofen—first day (in mg)	0	200	134.36	100	57.43	130.83	57.97	141.94	55.95	1.26	0.19
Ketoprofen—second day (in mg)	0	200	97.44	100	56.93	95.49	57.56	101.61	55.79	−0.70	−0.11
Acetaminophen—first day (in mg)	0	4000	2210.26	2000	800.71	2248.12	829.44	2129.03	735.16	−0.97	−0.15
Acetaminophen—second day (in mg)	0	3000	1497.44	2000	851.77	1511.28	840.42	1467.74	881.83	−0.33	−0.05

*Note*: *** *p* < 0.001.

**Table 2:** Correlations between variables measured in the study.

Variable	1		2		3	4		5	6		7		8	
1. Age	-													
2. BMI	0.03		-											
3. Diabetes (1 = yes, 0 = no)	0.13	*	0.15	*	-									
4. Morphine—first day	0.02		0.02		0.11	-								
5. Morphine—second day	0.03		−0.03		0.04	0.01		-						
6. Ketoprofen—first day	0.14		0.03		0.07	−0.08		−0.05	-					
7. Ketoprofen—second day	−0.16	*	0.05		0.02	0.21	**	0.02	0.04		-			
8. Acetaminophen—first day	−0.02		0.08		−0.02	0.14	*	−0.09	0.28	***	0.17	*		
9. Acetaminophen—second day	0.04		0.03		−0.08	0.13		0.00	−0.07		0.13		0.23	**

*Note*: *** *p* < 0.001, ** *p* < 0.01, * *p* < 0.05; Person correlations for continuous variables, tau-b Kendall correlation for diabetes.

**Table 3:** Multilevel regression of the doses of analgesics (standardized within substance) predicted from the diagnosis of diabetes, polymorphisms of *OPRM1*rs1799971, and substance administered.

	Step 1					Step 2				
Predictors	β	Post *SD*	95% CI		*p*	β	Post *SD*	95% CI		*p*
Age						0.00	0.03	−0.06	0.06	0.900
BMI						0.03	0.03	−0.02	0.10	0.260
Diabetes	0.03	0.05	−0.05	0.13	0.340	0.02	0.04	−0.06	0.09	0.740
Morphine	−0.03	0.06	−0.13	0.09	0.560	−0.03	0.05	−0.15	0.06	0.520
Ketoprofen	0.00	0.05	−0.10	0.08	0.999	0.00	0.06	−0.10	0.13	0.880
*OPRM1*rs1799971	−0.05	0.05	−0.13	0.03	0.280	−0.03	0.04	−0.11	0.03	0.400
Diabetes × Morphine	−0.08	0.06	−0.19	0.04	0.240	−0.07	0.05	−0.18	0.03	0.140
Diabetes × Ketoprofen	0.01	0.05	−0.09	0.12	0.800	0.02	0.06	−0.08	0.13	0.760
Diabetes × *OPRM1*rs1799971	0.02	0.05	−0.07	0.12	0.540	0.02	0.05	−0.08	0.12	0.520
Morphine × *OPRM1*rs1799971	−0.10	0.07	−0.22	0.03	0.100	−0.10	0.05	−0.22	0.00	0.060
Ketoprofen × *OPRM1*rs1799971	−0.05	0.06	−0.14	0.07	0.440	−0.04	0.06	−0.16	0.06	0.440
Diabetes × Morphine × *OPRM1*rs1799971	−0.21	0.06	−0.31	−0.11	0.001	−0.20	0.06	−0.31	−0.10	0.001
Diabetes × Ketoprofen × *OPRM1*rs1799971	−0.05	0.05	−0.16	0.04	0.260	−0.05	0.06	−0.19	0.06	0.340

*Note*: Post *SD*: Posterior Standard Deviation; 95% CI: 95% Credibility Interval; Diabetes: yes vs. no; Morphine: morphine vs. acetaminophen; Ketoprofen: ketoprofen vs. acetaminophen; *OPRM1*rs1799971: AA vs. AG/GG, AA *n* = 157, AG/GG *n* = 37.

**Table 4:** Multilevel regression of the doses of analgesics (standardized within substance) predicted from the diagnosis of diabetes, polymorphisms of *COMT* rs4633, and substance administered.

	Step 1					Step 2				
Predictors	β	Post *SD*	95% CI		*p*	β	Post *SD*	95% CI		*p*
Age						−0.01	0.03	−0.06	0.05	0.820
BMI						0.03	0.03	−0.03	0.09	0.320
Diabetes	−0.01	0.04	−0.09	0.06	0.720	−0.03	0.04	−0.10	0.05	0.580
Morphine	0.05	0.04	−0.04	0.12	0.260	0.04	0.04	−0.04	0.14	0.340
Ketoprofen	0.05	0.05	−0.05	0.14	0.320	0.05	0.04	−0.03	0.12	0.260
rs4633 CT	0.11	0.05	0.00	0.18	0.040	0.10	0.05	0.00	0.18	0.040
rs4633 TT	0.05	0.05	−0.04	0.13	0.400	0.06	0.05	−0.05	0.14	0.360
Diabetes × Morphine	0.08	0.05	0.00	0.17	0.080	0.08	0.04	−0.02	0.16	0.080
Diabetes × Ketoprofen	0.08	0.04	−0.01	0.14	0.080	0.07	0.05	−0.02	0.19	0.140
Diabetes × *COMT* rs4633 CT	0.12	0.04	0.02	0.19	0.020	0.11	0.05	0.03	0.20	0.020
Diabetes × *COMT* rs4633 CC	0.02	0.05	−0.11	0.10	0.720	0.02	0.05	−0.09	0.10	0.680
Morphine × *COMT* rs4633 CT	−0.03	0.06	−0.14	0.08	0.600	−0.02	0.06	−0.13	0.09	0.740
Morphine × *COMT* rs4633 CC	−0.05	0.07	−0.20	0.10	0.380	−0.06	0.07	−0.19	0.09	0.300
Ketoprofen × *COMT* rs4633 CT	−0.02	0.05	−0.13	0.07	0.560	−0.02	0.06	−0.14	0.08	0.720
Ketoprofen × *COMT* rs4633 CC	0.04	0.06	−0.07	0.16	0.460	0.04	0.06	−0.07	0.16	0.500
Diabetes × Morphine × *COMT* rs4633 CT	−0.06	0.06	−0.17	0.06	0.340	−0.06	0.05	−0.17	0.04	0.260
Diabetes × Morphine × *COMT* rs4633 CC	−0.07	0.06	−0.18	0.06	0.300	−0.08	0.06	−0.21	0.04	0.240
Diabetes × Ketoprofen × *COMT* rs4633 CT	−0.04	0.06	−0.18	0.07	0.320	−0.06	0.06	−0.17	0.04	0.440
Diabetes × Ketoprofen × *COMT* rs4633 CC	−0.03	0.06	−0.13	0.11	0.720	−0.03	0.07	−0.16	0.10	0.700

*Note*: Post *SD*: Posterior Standard Deviation; 95% CI: 95% Credibility Interval; Diabetes: yes vs. no; Morphine: morphine vs. acetaminophen; Ketoprofen: ketoprofen vs. acetaminophen; *COMT* rs4633 CT: CT vs. TT; *COMT* rs4633 CC: CC vs. TT: CC *n =* 50, CT *n =* 88, TT *n =* 57.

**Table 5:** Multilevel regression of the doses of analgesics predicted from the diagnosis of diabetes, polymorphisms of *COMT* rs4680, and substance administered.

	Step 1					Step 2				
Predictors	β	Post *SD*	95% CI		*p*	β	Post *SD*	95% CI		*p*
Age						0.00	0.03	−0.06	0.05	0.920
BMI						0.03	0.03	−0.03	0.09	0.340
Diabetes	−0.01	0.04	−0.09	0.06	0.840	−0.02	0.04	−0.10	0.05	0.680
Morphine	0.05	0.04	−0.04	0.12	0.280	0.04	0.04	−0.05	0.14	0.360
Ketoprofen	0.05	0.05	−0.05	0.14	0.280	0.06	0.04	−0.02	0.12	0.200
rs4680 AG	0.11	0.04	0.00	0.18	0.040	0.10	0.05	0.00	0.18	0.040
rs4680 GG	0.04	0.05	−0.05	0.12	0.440	0.05	0.05	−0.05	0.13	0.400
Diabetes × Morphine	0.08	0.05	−0.01	0.17	0.100	0.08	0.04	−0.02	0.16	0.080
Diabetes × Ketoprofen	0.08	0.04	−0.01	0.14	0.080	0.08	0.05	−0.02	0.19	0.140
Diabetes × *COMT* rs4680 AG	0.11	0.04	0.01	0.18	0.020	0.11	0.04	0.03	0.19	0.020
Diabetes × *COMT* rs4680 GG	0.02	0.05	−0.12	0.09	0.700	0.01	0.05	−0.09	0.10	0.720
Morphine × *COMT* rs4680 AG	−0.02	0.06	−0.13	0.09	0.740	−0.01	0.05	−0.12	0.09	0.860
Morphine × *COMT* rs4680 GG	−0.05	0.07	−0.20	0.12	0.560	−0.05	0.07	−0.18	0.10	0.420
Ketoprofen × *COMT* rs4680 AG	−0.01	0.05	−0.12	0.08	0.740	−0.01	0.06	−0.13	0.09	0.820
Ketoprofen × *COMT* rs4680 GG	0.06	0.06	−0.04	0.19	0.220	0.06	0.06	−0.05	0.18	0.220
Diabetes × Morphine × *COMT* rs4680 AG	−0.05	0.06	−0.16	0.06	0.360	−0.05	0.05	−0.16	0.04	0.360
Diabetes × Morphine × *COMT* rs4680 GG	−0.06	0.06	−0.18	0.08	0.440	−0.07	0.06	−0.20	0.05	0.340
Diabetes × Ketoprofen × *COMT* rs4680 AG	−0.04	0.06	−0.17	0.08	0.440	−0.05	0.06	−0.15	0.05	0.560
Diabetes × Ketoprofen × *COMT* rs4680 GG	−0.01	0.06	−0.11	0.12	0.880	−0.01	0.07	−0.15	0.12	0.840

*Note*: Post *SD*: Posterior Standard Deviation; 95% CI: 95% Credibility Interval; Diabetes: yes vs. no; Morphine: morphine vs. acetaminophen; Ketoprofen: ketoprofen vs. acetaminophen; *COMT* rs4680 AG: AG vs. AA; *COMT* rs4680 GG: GG vs. AA; AA *n =* 57, AG *n =* 89, GG *n =* 49.

**Table 6:** Multilevel regression of the doses of analgesics (standardized within the substance) predicted from the diagnosis of diabetes, polymorphisms of *COMT* rs4818, and substance administered.

	Step 1					Step 2				
Predictors	β	Post *SD*	95% CI		*p*	β	Post *SD*	95% CI		*p*
Age						0.00	0.03	−0.07	0.07	0.900
BMI						0.03	0.03	−0.02	0.09	0.200
Diabetes	−0.02	0.04	−0.11	0.06	0.620	−0.04	0.04	−0.11	0.05	0.420
Morphine	0.02	0.05	−0.08	0.10	0.780	0.01	0.05	−0.09	0.12	0.780
Ketoprofen	0.02	0.05	−0.08	0.13	0.620	0.03	0.05	−0.06	0.11	0.520
rs4818 CG	0.02	0.04	−0.07	0.09	0.520	0.02	0.04	−0.08	0.07	0.800
rs4818 GG	−0.03	0.06	−0.12	0.08	0.720	−0.02	0.06	−0.13	0.07	0.720
Diabetes × Morphine	0.06	0.05	−0.03	0.16	0.220	0.06	0.05	−0.05	0.15	0.260
Diabetes × Ketoprofen	0.05	0.05	−0.04	0.12	0.340	0.05	0.06	−0.06	0.18	0.400
Diabetes × *COMT* rs4818 CG	0.03	0.04	−0.07	0.10	0.420	0.02	0.04	−0.05	0.11	0.520
Diabetes × *COMT* rs4818 GG	−0.07	0.06	−0.23	0.01	0.080	−0.09	0.05	−0.20	0.01	0.100
Morphine × *COMT* rs4818 CG	−0.03	0.05	−0.14	0.06	0.540	−0.03	0.05	−0.13	0.07	0.560
Morphine × *COMT* rs4818 GG	−0.10	0.08	−0.23	0.07	0.280	−0.11	0.08	−0.23	0.09	0.200
Ketoprofen × *COMT* rs4818 CG	0.04	0.04	−0.05	0.13	0.300	0.04	0.05	−0.04	0.14	0.260
Ketoprofen × *COMT* rs4818 GG	0.01	0.07	−0.10	0.16	0.720	0.02	0.07	−0.11	0.16	0.800
Diabetes × Morphine × *COMT* rs4818 CG	−0.06	0.05	−0.15	0.04	0.300	−0.06	0.05	−0.15	0.03	0.280
Diabetes × Morphine × *COMT* rs4818 GG	−0.08	0.07	−0.21	0.06	0.420	−0.08	0.08	−0.22	0.05	0.300
Diabetes × Ketoprofen × *COMT* rs4818 CG	−0.01	0.05	−0.12	0.10	0.860	−0.01	0.05	−0.12	0.07	0.820
Diabetes × Ketoprofen × *COMT* rs4818 GG	−0.02	0.07	−0.14	0.10	0.620	−0.03	0.08	−0.20	0.11	0.760

*Note*: Post *SD*: Posterior Standard Deviation; 95% CI: 95% Credibility Interval; Diabetes: yes vs. no; Morphine: morphine vs. acetaminophen; Ketoprofen: ketoprofen vs. acetaminophen; *COMT* rs4818 CG: CG vs. CC, *COMT* rs4818 GG: GG vs. CC; CC *n* = 77, CG *n* = 95, GG *n* = 23.

**Table 7:** Multilevel regression of the doses of analgesics predicted from the diagnosis of diabetes, polymorphisms of *COMT* rs6269, and substance administered.

	Step 1					Step 2				
Predictors	β	Post *SD*	95% CI		*p*	β	Post *SD*	95% CI		*p*
Age						0.01	0.03	−0.05	0.06	0.780
BMI						0.03	0.03	−0.03	0.09	0.320
Diabetes	−0.04	0.04	−0.14	0.04	0.340	−0.06	0.04	−0.14	0.03	0.220
Morphine	0.02	0.05	−0.08	0.11	0.780	0.01	0.05	−0.09	0.13	0.800
Ketoprofen	0.02	0.05	−0.10	0.12	0.780	0.02	0.05	−0.07	0.10	0.660
rs4818 CG	0.02	0.04	−0.07	0.09	0.520	0.01	0.04	−0.07	0.09	0.800
rs4818 GG	−0.05	0.06	−0.15	0.07	0.440	−0.04	0.06	−0.16	0.06	0.540
Diabetes × Morphine	0.06	0.05	−0.04	0.17	0.320	0.06	0.05	−0.06	0.15	0.340
Diabetes × Ketoprofen	0.05	0.05	−0.05	0.12	0.420	0.05	0.06	−0.07	0.18	0.500
Diabetes × *COMT* rs6269 GA	0.03	0.04	−0.06	0.10	0.380	0.03	0.04	−0.04	0.11	0.480
Diabetes × *COMT* rs6269 GG	−0.10	0.06	−0.27	−0.01	0.020	−0.12	0.05	−0.23	−0.02	0.001
Morphine × *COMT* rs6269 GA	−0.04	0.05	−0.15	0.06	0.500	−0.03	0.05	−0.13	0.07	0.540
Morphine × *COMT* rs6269 GG	−0.10	0.08	−0.25	0.08	0.360	−0.11	0.08	−0.24	0.10	0.240
Ketoprofen × *COMT* rs6269 GA	0.05	0.04	−0.05	0.13	0.280	0.05	0.05	−0.04	0.14	0.220
Ketoprofen × *COMT* rs6269 GG	0.00	0.07	−0.13	0.15	0.960	0.00	0.07	−0.13	0.15	0.960
Diabetes × Morphine × *COMT* rs6269 GA	−0.06	0.05	−0.16	0.04	0.260	−0.06	0.05	−0.15	0.03	0.280
Diabetes × Morphine × *COMT* rs6269 GG	−0.08	0.08	−0.23	0.07	0.440	−0.08	0.08	−0.23	0.06	0.300
Diabetes × Ketoprofen × *COMT* rs6269 GA	−0.01	0.05	−0.12	0.10	0.860	−0.01	0.05	−0.11	0.07	0.820
Diabetes × Ketoprofen × *COMT* rs6269 GG	−0.03	0.07	−0.15	0.11	0.560	−0.04	0.09	−0.22	0.12	0.760

*Note*: Post *SD*: Posterior Standard Deviarion; 95% CI: 95% Credibility Interval; Diabetes: yes vs. no; Morphine: morphine vs. acetaminophen; Ketoprofen: ketoprofen vs. acetaminophen; *COMT* rs6269 GA: GA vs. AA, *COMT* rs6269 GG: GG vs. AA; AA *n* = 77, GA *n* = 95, GG *n* = 23.

## Data Availability

The data that support the findings of this study are available from the corresponding author upon reasonable request.

## Supplementary Materials

The following supporting information can be downloaded at: https://www.mdpi.com/article/10.3390/jcm14134634/s1, Table S1. Doses of analgesics depending on diagnosis of diabetes, polymorphisms of *OPRM1*rs1799971, and substance administered. Table S2. Doses of analgesics depending on diagnosis of diabetes, polymorphisms of *COMT* rs4633, and substance administered. Table S3. Doses of analgesics depending on diagnosis of diabetes, polymorphisms of *COMT* rs4680, and substance administered. Table S4. Doses of analgesics depending on diagnosis of diabetes, polymorphisms of *COMT* rs6269, and substance administered.
