# Supplementary material for: Do Diabetes and Genetic Polymorphisms in the COMT and OPRM1 Genes Modulate the Postoperative Opioid Demand and Pain Perception in Osteoarthritis Patients After Total Knee and Hip Arthroplasty?
Source: J Clin Med. 2025 Jun 30;14(13):4634. doi: 10.3390/jcm14134634 (PMC12249936; doi:10.3390/jcm14134634)
Supplement: Supplementary file 1 [file jcm-14-04634-s001.zip › jcm-3696190-supplementary.pdf]

Supplementary material

for

Do diabetes and genetic polymorphisms in the *COMT* and *OPRM1* genes modulate the postoperative opioid demand and pain perception in osteoarthritis patients after total knee and hip arthroplasty?

Table S1. Doses of analgesics depending on diagnosis of diabetes, polymorphisms of *OPRM1* rs1799971, and substance administered.

Diabetes	Alleles	n	N	Substance (in mg)	*M*	Mdn	*SD*	95% CI	
NO	AA	123	246	morphine	4.96	0	5.86	4.23	5.69
				ketoprofen	117.89	100	57.9	110.65	125.13
				acetaminophen	1934.96	2000	878.22	1825.21	2044.71
	AG+GG	31	62	morphine	5.81	0	7.31	3.99	7.63
				ketoprofen	101.61	100	61.38	86.33	116.89
				acetaminophen	1661.29	2000	848.21	1450.15	1872.43
YES	AA	34	68	morphine	6.76	10	6.79	5.15	8.37
				ketoprofen	120.59	100	65.92	104.92	136.26
				acetaminophen	1676.47	2000	984.07	1442.57	1910.37
	AG+GG	6	12	morphine	2.08	0	3.34	0.19	3.97
				ketoprofen	125	100	62.16	89.83	160.17
				acetaminophen	2166.67	2000	937.44	1636.26	2697.08

Note: *n*: number of participants; *N* = number of observations; 95%CI: 95% Confidence Interval for the mean

Table S2. Doses of analgesics depending on diagnosis of diabetes, polymorphisms of *COMT rs4633*, and substance administered.

Diabetes	Allele	*n*	*N*	Substance (in mg)	*M*	Mdn	*SD*	95%CI	
NO	CC	40	80	morphine	5.06	0	6.24	3.69	6.43
				ketoprofen	125	100	56.25	112.67	137.33
				acetaminophen	1887.5	2000	899.98	1690.28	2084.72
	CT	67	134	morphine	5.26	0	6.14	4.22	6.3
				ketoprofen	111.19	100	55.74	101.75	120.63
				acetaminophen	1843.28	2000	857.12	1698.15	1988.41
	TT	47	94	morphine	5	0	6.22	3.74	6.26
				ketoprofen	110.64	100	64.7	97.56	123.72
				acetaminophen	1925.53	2000	894.9	1744.62	2106.44
YES	CC	10	20	morphine	4	0	5.98	1.38	6.62
				ketoprofen	125	100	63.87	97.01	152.99
				acetaminophen	1650	2000	745.16	1323.42	1976.58
	CT	21	42	morphine	7.26	10	7.26	5.06	9.46
				ketoprofen	126.19	100	62.7	107.23	145.15
				acetaminophen	1976.19	2000	999.71	1673.84	2278.54
	TT	10	20	morphine	5.5	10	5.1	3.26	7.74
				ketoprofen	105	100	68.63	74.92	135.08
				acetaminophen	1400	1000	1046.3	941.44	1858.56

Note: *n*: number of participants; *N* = number of observations; 95%CI: 95% Confidence Interval for the mean

Table S3. Doses of analgesics depending on diagnosis of diabetes, polymorphisms of *COMT rs4680*, and substance administered.

Diabetes	Allele	*n*	*N*	Substance (in mg)	*M*	Mdn	*SD*	95%CI	
NO	AA	39	78	morphine	4.94	0	6.06	3.6	6.28
				ketoprofen	125.64	100	56.83	113.03	138.25
				acetaminophen	1871.79	2000	902.51	1671.5	2072.08
	GA	68	136	morphine	5.33	0	6.24	4.28	6.38
				ketoprofen	111.03	100	55.34	101.73	120.33
				acetaminophen	1852.94	2000	856.6	1708.97	1996.91
	GG	47	94	morphine	5	0	6.22	3.74	6.26
				ketoprofen	110.64	100	64.7	97.56	123.72
				acetaminophen	1925.53	2000	894.9	1744.62	2106.44
YES	AA	9	18	morphine	3.89	0	6.08	1.08	6.7
				ketoprofen	127.78	100	66.91	96.87	158.69
				acetaminophen	1611.11	2000	777.54	1251.9	1970.32
	GA	21	42	morphine	7.26	10	7.26	5.06	9.46
				ketoprofen	126.19	100	62.7	107.23	145.15
				acetaminophen	1976.19	2000	999.71	1673.84	2278.54
	GG	11	22	morphine	5.45	10	5.1	3.32	7.58
				ketoprofen	104.55	100	65.3	77.26	131.84
				acetaminophen	1454.55	1500	1010.76	1032.18	1876.92

Note: *n*: number of participants; *N* = number of observations; 95%CI: 95% Confidence Interval for the mean

Table S4. Doses of analgesics depending on diagnosis of diabetes, polymorphisms of *COMT* rs6269, and substance administered.

Diabetes	Allele	*n*	*N*	Substance (in mg)	*M*	Mdn	*SD*	95%CI	
NO	AA	61	122	morphine	5.16	0	6.1	4.08	6.24
				ketoprofen	109.84	100	63.52	98.57	121.11
				acetaminophen	1918.03	2000	886.91	1760.65	2075.41
	GA	74	148	morphine	5	0	6.23	4	6
				ketoprofen	115.54	100	55.59	106.58	124.5
				acetaminophen	1810.81	2000	867.8	1671	1950.62
	GG	19	38	morphine	5.53	2.5	6.34	3.51	7.55
				ketoprofen	126.32	100	55.43	108.7	143.94
				acetaminophen	2026.32	2000	884.91	1744.96	2307.68
YES	AA	16	32	morphine	7.19	10	6.21	5.04	9.34
				ketoprofen	112.5	100	70.71	88	137
				acetaminophen	1687.5	2000	1029.8	1330.69	2044.31
	GA	21	42	morphine	5.83	0	6.98	3.72	7.94
				ketoprofen	130.95	100	60.44	112.67	149.23
				acetaminophen	1833.33	2000	985.67	1535.23	2131.43
	GG	4	8	morphine	2.5	0	4.63	-0.71	5.71
				ketoprofen	100	100	53.45	62.96	137.04
				acetaminophen	1625	2000	744.02	1109.42	2140.58

Note: *n*: number of participants; *N* = number of observations; 95%CI: 95% Confidence Interval for the mean
